# Tenascin-C in Osteoarthritis and Rheumatoid Arthritis

**DOI:** 10.3389/fimmu.2020.577015

**Published:** 2020-09-30

**Authors:** Masahiro Hasegawa, Toshimichi Yoshida, Akihiro Sudo

**Affiliations:** ^1^ Department of Orthopaedic Surgery, Mie University Graduate School of Medicine, Tsu, Japan; ^2^ Department of Pathology & Matrix Biology, Mie University Graduate School of Medicine, Tsu, Japan

**Keywords:** tenascin-C, osteoarthritis, rheumatoid arthritis, cartilage, repair, synovitis, animal model

## Abstract

Tenascin-C (TNC) is a large multimodular glycoprotein of the extracellular matrix that consists of four distinct domains. Emerging evidence suggests that TNC may be involved in the pathogenesis of osteoarthritis (OA) and rheumatoid arthritis (RA). In this review, we summarize the current understanding of the role of TNC in cartilage and in synovial biology, across both OA and RA. TNC is expressed in association with the development of articular cartilage; the expression decreases during maturation of chondrocytes and disappears almost completely in adult articular cartilage. TNC expression is increased in diseased cartilage, synovium, and synovial fluid in OA and RA. In addition, elevated circulating TNC levels have been detected in the blood of RA patients. Thus, TNC could be used as a novel biochemical marker for OA and RA, although it has no specificity as a biochemical marker for these joint disorders. In a post-traumatic OA model of aged joints, TNC deficiency was shown to enhance cartilage degeneration. Treatment with TNC domains results in different, domain-specific effects, which are also dose-dependent. For instance, some TNC fragments including the fibrinogen-like globe domain might function as endogenous inducers of synovitis and cartilage matrix degradation through binding with toll-like receptor-4, while full-length TNC promotes cartilage repair and prevents the development of OA without exacerbating synovitis. The TNC peptide TNIIIA2 also prevents cartilage degeneration without causing synovial inflammation. The clinical significance of TNC effects on cartilage and synovium is unclear and understanding the clinical significance of TNC is not straightforward.

## Introduction

Osteoarthritis (OA) is a well-known cause of disability, with an estimated global prevalence of more than 30% (1). Further, its prevalence is increasing because of rapid population aging. Risk factors for OA include person factors (age, sex, obesity, and genetics) and joint factors (deformity, malalignment, and injury) that interact in a complex manner (2). Rheumatoid arthritis (RA) is a chronic inflammatory disease that can cause joint destruction as well as disability (3). The global prevalence of RA is around 1%, and genetics are the principal risk factor for developing RA, while smoking is the main environmental risk factor. RA is most typically found in elderly women (3, 4). The pathogenesis of RA and periodontitis might be similar, with both diseases involving chronic inflammation and bone erosion (5).

Tenascin-C (TNC) is a non-structural extracellular matrix (ECM) protein that is highly expressed in morphogenesis and tissue remodeling, and has many effects on cellular responses (6). Emerging evidence suggests that TNC might be involved in the pathogenesis of OA and RA. In this review, we present the current understanding of the role of TNC in cartilage and in synovial biology, across both OA and RA.

## Structure and Distribution of Tenascin-C

TNC is a hexameric glycoprotein component of the ECM. The TNC molecule is composed of large molecular weight subunits (220–400 kDa) consisting of four distinct domains, with each TNC subunit consisting of a tenascin assembly (TA) domain that forms a coil at the N-terminus, 14.5 epidermal growth factor-like (EGF-L) repeats, up to 17 fibronectin type III (FNIII) -like repeats, and a C-terminal fibrinogen-like globe (FBG) domain. The FNIII-like repeats undergo alternative splicing to bind different ECM proteins, such as fibronectin, syndecan-4, and integrins αVβ3 and α8β1 (7–10). Furthermore, the FNIII-like repeats bind to a number of growth factors including fibroblast growth factor (FGF), platelet-derived growth factor (PDGF), and the transforming growth factor-β (TGF-β) family. The FBG domain binds to αVβ3 integrin and receptor-type tyrosine-protein phosphatase zeta, and activates toll-like receptor-4 (TLR4) (9, 11). TNC can drive a range of processes including cell migration, attachment, proliferation, and synthesis of proteases and proinflammatory cytokines (9). Growth factors, such as TGF-β, FGF, and PDGF, can induce TNC expression. TNC is transiently expressed in the mesenchyme around developing organs, such as mammary glands, teeth, and kidneys. TNC is also expressed during embryo development in cartilage, ligament, tendon, periosteum, myotendinous junction, smooth muscle, and perichondrium. Expression of TNC is generally low in adult tissues, but is transiently elevated following tissue injury. Once the damaged tissue is repaired, TNC expression is inhibited. TNC shares a structural relationship with fibronectin (12); although fibronectin is adhesive in nature while TNC is only weakly adhesive (13). Functional inhibition of syndecan-4 suppresses TNC activity. In contrast, overexpression of syndecan-4 neutralizes the effect of TNC. Thus TNC and syndecan-4 work together to control fibroblast signaling and morphology, and to regulate the contraction of the matrix, including tissue repair (14, 15). TNC expression is regulated by mechanical stress, and is elevated in tissues that experience high tensile stress, such as smooth muscle, ligaments, and tendons (16).

## Cartilage

Articular cartilage is an aneural, avascular, alymphatic and viscoelastic tissue. It’s extremely low coefficient of friction contributes to the lubrication of joint movement. Water accounts for up to 85% of the wet weight of cartilage (17). By dry weight, the large aggregating proteoglycan aggrecan and type II collagen are the main ECM components of cartilage (18). Chondrocytes produce ECM with scant cell turnover (19). During the first stage of joint development, an interzone emerges at the presumptive joint site. Joint cavitation subsequently occurs at the center of the interzone, and cells within the interzone form the joint and cartilage (20). TNC participates in chondrogenesis and cartilage development (7, 8). At the early stage of articular cartilage formation the Indian hedgehog (Ihh), Erg, noggin, Wnt9a, and Gdf5 genes are expressed strongly. At the later stage, the expression of these early regulatory genes is downregulated, and the expression of structural genes, including proteoglycan (Prg) 4, type II collagen, CD44, and TNC, becomes preponderant (20, 21). In comparison, TGF-β, FGF18, and parathyroid hormone-related protein are continuously expressed during articular cartilage formation (20). In the newborn mouse knee, articular cartilage is a thin, dense tissue consisting of small, randomly oriented Prg4- and TNC-expressing cells (22). In mature cartilage, TNC is only present in the perichondrium (23), and disappears almost completely in adult articular cartilage (24). When the articular cartilage of TNC-knockout mice at a postnatal age of 8 weeks was compared to that of age-matched wild-type (WT) mice, the tangential/transitional zone was thicker and the density of chondrocytes was lower in WT mice than in the TNC-knockout mice. This observation in mice implies that TNC plays a role in increasing articular cartilage volume as well as producing ECM from birth to 2 months of age (25).

## Cartilage Repair

Articular cartilage has a limited potential for repair and damaged cartilage is associated with the development of OA. Many strategies to repair cartilage have been proposed including bone marrow stimulation techniques, osteochondral graft, cell-based cartilage repair procedures, and the use of growth factors, such as TGF-β, bone morphogenetic protein-2, and FGF (26, 27). Autologous chondrocyte implantation offers great promise with good long-term results; however, its applicability for large defects is limited (28). Although local administration of growth factors could be easily implemented, this strategy has not been used in clinical trials (26). Notably, TNC appears to be capable of mediating repair in human OA cartilage *in vitro* (29). During *in vivo* cartilage repair, TNC expression was found at the early phase with expression disappearing at the late phase with cartilage maturation (30). In TNC-knockout BALB/c mice, cartilage repair was found to be significantly delayed compared to WT mice, and the deficiency of TNC accelerated degeneration of cartilage (30). When examining the effects of intra-articular TNC administration on the repair of full-thickness cartilage defects in rabbits using scaffolding matrices, full-length TNC (10 µg/mL) was found to promote the repair of cartilage *in vivo* (27). However, scaffold impregnated with a higher concentration (100 µg/mL) of TNC did not facilitate cartilage repair. In a BALB/c mouse model, full-length TNC (100 µg/mL) promoted cartilage repair in the absence of scaffold (31) (**Table 1**).

**Table 1:** Effect of tenascin-C domain on cartilage and synovial responses.

Tissue	Addition of TNC	Domain	Response	Cell type/species	Reference
Cartilage	*in vitro*	EGF-L domain and FNIII 3–8	Aggrecan-degrading ability	Human chondrocyte	Sofat et al. (32)
		TNIIIA2	Upregulating TNF-α, MMP-3, bFGF	Human chondrocyte	Hattori et al. (33)
		Full-length	Inducing IL-6, PGE2, nitrate release, upregulating ADAMTS4	Bovine and human chondrocytes	Patel et al. (34)
		Full-length	Cartilage proliferation	Human chondrocyte	Nakoshi et al. (29)
		Full-length	Upregulating TNF-α, IL-1β, ADAMTS4, MMP-3, MMP-13, TGF-β, TIMP3	Human chondrocyte	Unno et al. (31)
			Downregulating ADAMTS5		
	*in vivo*	FBG	Inducing cartilage proteoglycan loss	129/SV mouse	Midwood et al. (35)
		TNIIIA2	Preventing cartilage degeneration	BALB/c mouse	Hattori et al. (33)
		Full-length	Preventing cartilage degeneration	BALB/c mouse	Matsui et al. (36)
		Full-length	Repairing cartilage defects	Japanese white rabbit	Ikemura et al. (27)
		Full-length	Repairing cartilage defects	BALB/c mouse	Unno et al. (31)
Synovium	*in vitro*	Full-length	Upregulating IL-6	Human synovial fibroblast	Midwood et al. (35)
		FBG			
		FNIII 3	Upregulating TNF-α, IL-1α, IL-1β, IL-6, CCL2, CCL3, CCL4, CXCL2, CXCL5, CXCL12, MMP-9	Mouse synovial macrophage, Mouse synovial fibroblast	Kanayama et al. (37)
			Downregulating MMP-2		
	*in vivo*	FBG	Inducing synovitis	129/SV mouse	Midwood et al. (35)
		FNIII 1-5	Antibody directed against FNIII1-5 reducing synovitis	BALB/c mouse	Mehta et al. (38)
		TNIIIA2	No enhancement of synovitis	BALB/c mouse	Hattori et al. (33)
		Full-length	No enhancement of synovitis	BALB/c mouse	Unno et al. (31)

TNC, tenascin-C; EGF-L, epidermal growth factor–like; FNIII, fibronectin type III; FBG, fibrinogen-like globe; TNF, tumor necrosis factor.

MMP, matrix metalloproteinase; FGF, fibroblast growth factor; IL, interleukin; PGE, prostaglandin E.

ADAMTS, a disintegrin and metalloproteinase with thrombospondin motifs; TGF, transforming growtfactor.

TIMP, tissue inhibitor of metalloprotease; CCL, chemokine (C-C motif) ligand; CXCL, chemokine (C-X-C motif) ligand.

## Osteoarthritis

Pathologic alterations in cartilage, bone, synovium, ligament, and meniscus are observed in OA, revealing OA to be a whole joint disease, with synovitis being one of the common features of OA. Synoviocytes synthesize hyaluronic acid and lubricin, which contribute to normal joint function (39). During progression of OA, the synovium can be a source of matrix metalloproteinase (MMP) and aggrecanase, which contributes to the degradation of the cartilage matrix. OA has historically been categorized as a non-inflammatory form of arthritis; however, the role of the development of synovitis in OA pathogenesis has been demonstrated (40–42). The synovium can produce soluble inflammatory mediators, including cytokines and chemokines, that are detected in joint tissues and synovial fluid in OA, and contribute to cartilage degeneration (40). Animal models of OA have been categorized into spontaneous and induced models, and the post-traumatic OA (PTOA) model is the most widely studied. Methods for inducing PTOA models include anterior cruciate ligament transection, medial collateral ligament transection, meniscectomy, and destabilization of the medial meniscus. In chemically induced models, sodium monoiodoacetate (MIA) is most commonly used to induce OA (43).

In clinical practice, the severity of OA is generally assessed with plain radiographs (44). Biochemical markers, however, provide an opportunity to better diagnose and stratify patients. Although cartilage oligomeric matrix protein in serum and C-terminal telopeptide of collagen type II in urine are the most widely investigated markers of tissue degradation, no marker has been well validated in clinical use for the diagnosis and monitoring of OA (39). It is not possible to determine the site of origin when biochemical markers are measured in serum or urine, but markers present in synovial fluid can provide insight into the damage in an individual joint (39). TNC levels in the synovial fluid were shown to be significantly increased in patients with knee OA (34, 45). In addition, TNC levels correlated with the radiographic grading levels (45), and TNC in the synovial fluid has been demonstrated to be a useful marker of OA progression (45). In a canine PTOA model, TNC levels increased markedly during the acute phase and then decreased over time, but remained elevated relative to the control group, even after 12 months (46).

**Figure 1:**
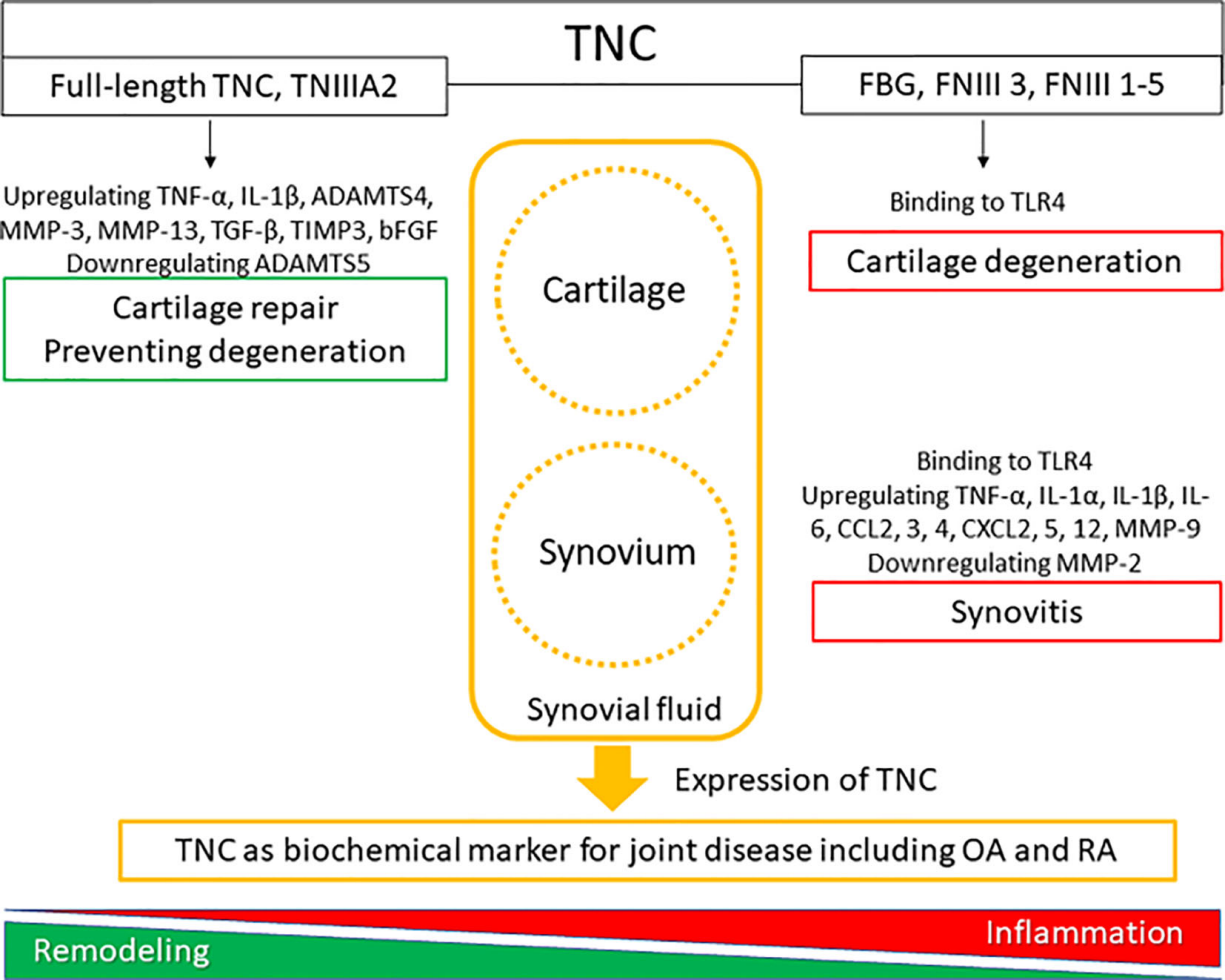
The roles of tenascin-C (TNC) in synovial joint biology. TNC expression is upregulated in degenerated cartilage and inflamed synovial tissue.

Immunohistochemical analysis of TNC expression revealed that TNC staining intensity increased with the degeneration of cartilage in comparison with normal cartilage (29). TNC staining is observed on the OA cartilage surface overlying chondroitin sulfate (CS)-positive areas (29), and enhanced TNC staining is associated with clusters of chondrocytes (47). These results suggest that the distribution of TNC is correlated with CS production and chondrocyte proliferation in OA cartilage. In cultured human OA chondrocytes, treatment with TNC induced chondrocyte proliferation and increased aggrecan levels (29). Tumor necrosis factor (TNF)-α stimulates TNC expression through nuclear factor-κB signaling with RelA subunit activation, which could affect cell proliferation. TNC is reported to have a potential role in remodeling of cartilage (47); however, elevated levels of TNC could induce inflammatory mediators and promote degradation of matrix in OA cartilage (34). TNC plays dual roles in synovial fluid, where it not only acts as a marker of joint damage, but also stimulates joint degradation (46). These two opposing roles of TNC in synovial fluid may stem from the versatile nature of this glycoprotein (48). Full-length TNC prevented cartilage degeneration in a PTOA model using BALB/c mice (36). In contrast, recombinant TNC fragments induced aggrecanase activity and mediated cartilage degeneration. The EGF-L and FNIII-like domains 3–8 of TNC showed high aggrecan-degrading activity, which was not observed with either full-length TNC or other TNC domains (32) (**Table 1**, **Figure 1**). TNIIIA2 is a 22-mer peptide of TNC that induces β1 integrin activation through syndecan-4, and intra-articular injection of TNIIIA2 prevented degeneration of articular cartilage in a PTOA model using BALB/c mice (33). The inflammatory effects of TNC were identified through binding with TLR4, and integrins α9β1 and αVβ3 (49), but further work is required to clarify whether TNC contributes to OA pathogenesis *via* integrins. In cultured human and bovine chondrocytes, treatment with TNC upregulated interleukin (IL)-6, prostaglandin E_2_, nitrate release, and disintegrin and metalloproteinase with thrombospondin motifs-4 (34). Treatment with TNC decreased the amount of proteoglycan present in cartilage explants (32, 34). The FBG domain was reported to be an endogenous inducer of cartilage matrix degradation (32), while full-length TNC did not cause the severe inflammation observed with the FBG domain (27, 31, 36). Animal OA models using TNC-knockout mice have generated mixed results. Intra-articular injection of TNC induced synovitis in a TLR4-dependent manner using 129/SV mice (35) (**Table 1**). TLR4 activates the Fcγ receptor, and regulate the early onset of joint inflammation and cartilage damage during immune complex-mediated arthritis (50). TNC is not involved in the early onset of joint inflammation but is required for maintenance of inflammatory processes (35). The damage-associated molecular patterns CD14 ligand appears to contribute to cartilage repair in OA (51), although CD14 is a modulator of innate inflammatory signaling that acts as a receptor for bacterial lipopolysaccharide with TLR4 (52). There are reports of beneficial effects for joint tissues (31, 36) in BALB/c mice, while deleterious effects for joint tissues were found in a different mouse species (129/SV).

Genetic variations might play a role in the differential responses in cartilage and synovium. For example, the MRL/MpJ strain mouse has an impaired inflammatory response and no susceptibility to OA (53). Moreover, MRL/MpJ mice showed lower levels of IL-1 and higher levels of IL-4 and IL-10 compared with C57BL/6 mice in a PTOA model (53). STR/ort mice are highly susceptible to OA, while C57BL/6J mice are only moderately susceptible (54).

## Rheumatoid Arthritis

RA is characterized by swelling, tenderness, and destruction of joints due to synovitis, inflammation, and autoantibodies, particularly to rheumatoid factor and citrullinated peptide, which can cause cartilage and bone damage and consequent disability (3, 4). The 2010 American College of Rheumatology/European League Against Rheumatism RA classification criteria are widely used for clinical diagnosis (55). The increased risk for RA in patients with the shared epitope is associated with seropositivity for autoantibodies against rheumatoid factor, which are autoantibodies against IgG and citrullinated peptide (ACPA). The inflammatory milieu in the synovial joint is regulated by a complex cytokine and chemokine network. TNF-α and IL-6 are essential to the process, whereas IL-1 and various chemokines may be less important (3, 56). Serum biomarkers are routinely used in monitoring of disease progression including erythrocyte sedimentation rate (ESR), C-reactive protein (CRP), and MMP-3 (57). Interaction of TNC and α9β1 integrin induced the expression of IL-6 and MMPs in synovial fibroblasts, and IL-1β and TNF-α in synovial macrophages (58).

Zymosan can be used to induce acute synovitis in 129/SV mice, where synovitis and cartilage proteoglycan loss were observed at 4 days in WT mice. In contrast, TNC-knockout mice did not exhibit any synovitis or loss of cartilage proteoglycan (35) (**Table 1**). TNC induced the synthesis of proinflammatory cytokines *via* endogenous activation of TLR4 (35). Using 129/SV mice, intra-articular injection of 1 or 3 µg FBG induced synovitis, inflammatory cell infiltration, pannus formation, and loss of cartilage proteoglycan; in contrast, 100 ng FBG injection did not induce any inflammation or proteoglycan loss (35) (**Table 1**). However, intra-articular administration of full-length TNC to BALB/c mice induced similar synovial inflammation compared to no TNC administration (31, 36). In a PTOA model using BALB/c mice, low-grade synovitis occurred at 2 weeks, but these changes improved at 4 weeks in mice with and without intra-articular injection of TNIIIA2 (33) (**Table 1**). While the FBG domain of TNC could be a critical driver of synovial inflammation in 129/SV mice, administration of full-length TNC and TNIIIA2 had no role in synovial inflammation in BALB/c mice (**Figure 1**). The capacity of TNC to exert both beneficial and deleterious effects on joint tissues is fascinating, and the mechanism underlying the dual nature of TNC remains poorly understood. Moreover, the dose of TNC administered appears to be an important factor in determining the *in vivo* effects of TNC.

TNC plays an important role in physiological tissue repair but also drives pathological inflammation and fibrosis (49). Intense TNC immunoreactivity was found in the RA synovium with strong chronic inflammation and fibrosis (59). Elevated circulating TNC levels have been detected in the blood of RA patients, and patients with late stage RA have higher TNC levels compared to those with early stage RA (60). TNC levels in blood and synovial fluid were not reported to correlate with CRP (59, 60). Moreover, blood levels of TNC did not correlate with other biomarkers, such as ESR and ACPA (60). However, TNC levels in blood correlated positively with erosion scores determined by ultrasound in early stage RA (60). TNC concentrations in synovial fluid were reported to be fourfold higher in RA compared with OA (59). Multiple citrullination sites exist in the FBG domain of TNC, and citrullinated TNC (cTNC) 5 was recognized as a biochemical marker that can be detected years before the onset of RA (61). Periodontitis is a risk factor for RA, and antibodies to cytokeratin 13 were found to correlate with anti-cTNC5 (62). The TN64 antibody is directed against the FNIII**-**like repeats 1-5 (TNfnIII 1-5) of TNC, and has been observed to prevent fibroblast-mediated cartilage destruction. The TN64 antibody was evaluated in collagen-induced arthritis in BALB/c mice, and TN64 was found to prevent the induction of arthritis by downregulation of TNF-α, IL-6, IL-10, IL-12, and IFN-γ (38). Both full-length TNC and the FBG domain induced synthesis of IL-6 in synovial fibroblasts (35). Mapping the active domain within TNC demonstrated a unique structural FBG epitope, essential for binding to and activating TLR4 (63). Monoclonal antibodies recognizing the FBG domain of TNC inhibited release of TNF-α, IL-6, and IL-8 by RA synovium (64). Blocking inflammatory signals from the ECM including TNC domains represents a potential novel therapeutic strategy for treating RA that may avoid global immune suppression.

It is not clear how the different domains in TNC interact with each other at a functional level and knowledge of different ligand binding modes of TNC is also limited (49). Moreover, it remains unclear whether activation of TLR4 by the FBG domain occurs in isolation from or in synergy with other TNC domains. Similarly, little is known about how tissue-specific responses to TNC are mediated (64). The clinical significance of TNC effects on cartilage and synovium is unclear and understanding the clinical significance of TNC is not straightforward, as it appears to contribute to both beneficial and deleterious effects in a context-dependent manner (65). However, TNC is clearly an important molecule involved in controlling cellular activity during tissue remodeling and inflammation (**Figure 1**). TNC does not have any specificity as a biochemical marker for OA and RA but has been shown to be a potential marker for other diseases. Elevated TNC expression was reported to predict poor prognosis among patients with various cancers, and TNC can be a serum biochemical marker for cancer (66). Serum TNC levels in patients with asthma was associated with clinical features of asthma, suggesting that serum TNC can be a biochemical for asthma (67). Circulating TNC levels were also elevated in patients with ankylosing spondylitis, systemic lupus erythematosus, psoriatic arthritis, ulcerative colitis, and Crohn’s disease (60, 68).

## Conclusion

TNC is a key molecule in tissue remodeling and is associated with OA and RA. TNC could be used as a novel biochemical marker for OA and RA, although it has no specificity as a biochemical marker for these joint disorders. Administration of TNC showed both beneficial and deleterious effects across different joint tissues and different TNC domains, and it’s *in vivo* effects were clearly dose-dependent. Full-length TNC and TNIIIA2 prevented the development of OA in a PTOA model using BALB/c mice, suggesting that TNC domains and animal species might influence the type and nature of the responses obtained in pre-clinical studies. Thus, the clinical significance of TNC effects on cartilage and synovium awaits further clarification.

## Author Contributions

MH and TY designed and reviewed the paper and contributed in drafting the manuscript. AS reviewed the manuscript. All authors contributed to the article and approved the submitted version.

## Conflict of Interest

The authors declare that the research was conducted in the absence of any commercial or financial relationships that could be construed as a potential conflict of interest.
